# Cytokine production pattern of T lymphocytes in neonatal arterial ischemic stroke during the first month of life—a case study

**DOI:** 10.1186/s12974-018-1229-y

**Published:** 2018-06-22

**Authors:** Anna Bajnok, László Berta, Csaba Orbán, Tivadar Tulassay, Gergely Toldi

**Affiliations:** 1 First Department of Obstetrics and Gynecology, Semmelweis University, Baross str. 27, Budapest, H-1088 Hungary; 2 First Department of Pediatrics, Semmelweis University, Bókay János str. 53-54, Budapest, H-1083 Hungary; 3 MTA-SE Pediatrics and Nephrology Research Group, Budapest, Hungary; 4 Neonatal Unit, Birmingham Women’s and Children’s Hospital, Birmingham, UK

**Keywords:** Neonatal arterial ischemic stroke, Hypoxic ischemic encephalopathy, Inflammatory response, Cytokine network, T lymphocytes

## Abstract

**Background:**

The perinatal period carries the highest risk for stroke in childhood; however, the pathophysiology is poorly understood and preventive, prognostic, and therapeutic strategies are not available. A new pathophysiological model describes the development of neonatal arterial ischemic stroke (NAIS) as the combined result of prenatal inflammation and hypoxic–ischemic insult. Neuroinflammation and a systemic inflammatory response are also important features of NAIS. Identifying key players of the inflammatory system is in the limelight of current research.

**Case presentation:**

We present four NAIS cases, in whom detailed analysis of intracellular and plasma cytokine levels are available from the first month of life. All neonates were admitted with the initial diagnosis of hypoxic ischemic encephalopathy (HIE); however, early MRI examination revealed NAIS. Blood samples were collected between 3 and 6 h of life, at 24 h, 72 h, 1 week, and 1 month of life. Peripheral blood mononuclear cells were assessed with flow cytometry and plasma cytokine levels were measured. Pooled data from the cohort of four NAIS patients were compared to infants with HIE.

At 6 and 72 h of age, the prevalence of IL10+ CD8+ lymphocytes remained lower in NAIS. At 6 h, CD8+ lymphocytes in NAIS produced more IL-17. At 72 h, CD8+ cells produced more IL-6 in severe HIE than in NAIS, but IL-6 production remained elevated in CD8 cells at 1 month in NAIS, while it decreased in HIE. At 1 week, the prevalence of TGF-β + lymphocytes prone to enter the CNS was elevated in NAIS. On the other hand, by 1 month of age, the prevalence of TGF-β + CD4+ lymphocytes decreased in NAIS compared to HIE. At 72 h, we found elevated plasma levels of IL-5, MCP-1, and IL-17 in NAIS. By 1 month, plasma levels of IL-4, IL-12, and IL-17 decreased in NAIS but remained elevated in HIE.

**Conclusions:**

Differences in the cytokine network are present between NAIS and HIE. CD8 lymphocytes appear to shift towards the pro-inflammatory direction in NAIS. The inflammatory response appears to be more pronounced at 72 h in NAIS but decreases faster, reaching lower plasma levels of inflammatory markers at 1 month.

## Background

The perinatal period carries the highest risk for stroke in the entire childhood, with almost similar incidence to that in the elderly population. “Perinatal stroke” is a broad term defining a group of heterogeneous conditions characterized by the focal disruption of cerebral blood flow, of which neonatal arterial ischemic stroke (NAIS) is one of the most common subtypes, with an incidence of 1/8000 live births. NAIS by definition is an arterial ischemic stroke, with clinical symptoms occurring in the neonatal period (within the first 28 days of life) which are supported by radiological evidence [1, 2]. NAIS generally occurs in term neonates and presents a major risk for life-long motor, cognitive, and/or behavioral disabilities ranging from fine motor impairments to unilateral cerebral palsy, which develops in around 20–30% of affected neonates. Thus NAIS is a leading cause of cerebral palsy [3].

Interestingly, in almost all cases of NAIS, the intra-cranial arteries developing from the carotid arterial tree are affected, i.e., the proximal parts of the anterior cerebral artery (ACA), the middle cerebral artery (MCA), and the posterior cerebral artery (PCA)—while the basilar artery and the extra-cranial arteries are left unaffected [4–6]. The area of the left MCA is the most common localization of the ischemic lesion, resulting in a higher incidence of right-sided congenital hemiplegia [1]. The clinical presentation of NAIS is often subtle and non-specific, the most frequent symptoms being seizures, general hypotonia, lethargy, and poor feeding, making the timely diagnosis of NAIS difficult [7]. Diagnosis of NAIS is often delayed, due to the prenatal onset or absence of specific signs; therefore, the primary focus regarding therapeutic interventions is focused on prevention and post-insult anti-inflammatory mechanisms [3]. Another challenge regarding the diagnosis of NAIS is the fact that the risk factors and clinical signs of global hypoxic-ischemic encephalopathy (HIE) due to perinatal asphyxia show a significant overlap with NAIS and the two often co-occur [7–9]. Differentiating between the two syndromes is a complex question, some studies list perinatal asphyxia as an independent risk factor for NAIS [10], while neuroinflammation following ischemia appears to be a common feature of the two.

The pathophysiology of NAIS is poorly understood, and disease-specific preventive measures, prognostic factors, and therapeutic strategies are not available. The risk factors of NAIS appear to be specific for the perinatal period and can be clearly distinguished from the risk factors of stroke in older children or adults. Major risk factors such as congenital heart disease or bacterial meningitis can be held accountable for approximately 30% of NAIS occurrences; however, in most of the cases, NAIS is thought to be multifactorial, and in 25–40% of cases, no identifiable risk factor is present [11, 12]. Only a few case-control studies have aimed to specify risk factors; however, the number of cases was relatively low and there is little consistency in the observed maternal, obstetric, and neonatal risk factors [7, 13, 14]. The only independent risk factor, which appeared in all case-control studies, was perinatal inflammation, while genetic prothrombotic disorders were not found to be associated with the frequency of NAIS [3].

According to the classic pathophysiological hypothesis, most ischemic lesions are a result of thromboembolic events, where the presumable source of the thrombi is the placenta or the umbilical vessels [15]. While in some instances this could be the case, this hypothesis does not give a plausible explanation to why NAIS is almost exclusively affecting the intracranial arterial territories developing from the carotid arterial tree, while the incidence of basilary arterial or extracerebral infarcts is negligible [15]. In addition, there is angiographic evidence indicating a possibility of local arterial wall defects and in situ thrombus generation [9, 16]. Based on these findings, Giraud et al. proposed a different pathomechanism, where maternofetal inflammation induces focal arteritis specific to the intracranial arteries developing from the carotid arterial tree, which are susceptible to NAIS [3]. Using a preclinical rat model of chorioamnionitis, they were able to demonstrate that classic prothrombotic stress applied alone to the MCA was not enough to induce NAIS; however, when combined with in utero exposure to inflammation-inducing agent (LPS), the same stress leads to the classical symptoms of NAIS and motor impairment. They also examined the walls of arteries susceptible to NAIS and found that the constitutive expression of certain pro-inflammatory cytokines, i.e., TNF-α and IL-1β, was higher in the susceptible intra-cerebral arteries compared to extra-cerebral arteries. Furthermore, pups born from LPS-exposed dams developed a specific cerebral arteritis with increased presence of inflammatory cells (macrophages) and elevated levels of pro-inflammatory cytokines IL-1β, TNF-α, and MCP-1 with increased IL-1/IL-1 receptor antagonist (ra) ratio in NAIS susceptible arteries, but not elsewhere. [5]

Based on preclinical findings, a new pathophysiological hypothesis is being raised, which considers the complex and bidirectional relationship of coagulation and inflammatory pathways [17]. It describes the development of NAIS as the result of a multiple hit mechanism, originating from both perinatal inflammation and hypoxia–ischemia (HI) [3]. Giraud et al. have identified three major risk factors, the combination of which could lead to NAIS: (i) materno-fetal inflammation leading to focal arteritis of arteries susceptible to NAIS, (ii) a window of susceptibility in the development of the neonatal brain, and (iii) the physiological pro-coagulatory state of the perinatal period aggravated by the inflammatory response [5]. Prenatal exposure to inflammation appears to sensitize the brain for postnatal HI injury [18–20]. The inflammation could be of maternal, placental, or fetal origin, which is in line with the proposed risk factors of NAIS (preeclampsia, chorioamnionitis, maternal fever, infection) [3, 10]. In addition, the initial hypoxic insult—as is now commonly recognized—is followed by a generalized neuroinflammatory response. The initial hypoxic insult leads to primary energy failure, necrosis, subsequent glutamate release, oxidative stress, and as a result excitotoxicity and widespread necrosis and necroptosis within the first 6 h of the insult [21–23]. Neural tissue damage could be aggravated by previous sensitization due to inflammation, which could lead to increased oxidative stress and an increased production of pro-inflammatory cytokines [19, 24–28]. The second phase of neuroinflammation occurs from 24 until 72 h after the initial HI insult and is characterized by the production of inflammatory cytokines, the migration of leukocytes into the brain tissue, and consequent apoptosis. By identifying the key players of the neuroinflammatory and apoptotic pathways, this second phase could be an important time for targeted intervention [3, 29, 30]. Inflammatory cells can be detected for weeks or even months following the HI insult [31], and this sustained inflammatory status can be considered the tertiary, latent phase of the inflammatory response, which could also be important regarding neurological outcome.

The primary aim of current research in the field of perinatal stroke is to gain a better understanding of the pathomechanism of the disease with specific regard to the activation of the inflammatory pathway that could present a possibility for more specific diagnosis, intervention, and even prevention [3]. Human experimental data on inflammatory markers is scarce; one case-control study is available, where they aimed to describe the levels of different cytokines in the plasma of NAIS patients. Elevated levels of TNF-α, IL-2, IL-6, and IL-8 were observed 6 months following acute ischemic stroke (AIS) compared to healthy controls. However, no differences were observed in soluble endothelial protein C receptor (sEPCR), IL-11, and FVIII median levels [32]. The limitation of this study is that they included both neonatal and pediatric stroke cases (from birth until 18 years of age), which are now viewed as clinically distinct syndromes. These results indicate that an ongoing inflammation could be observed up to 6 months following AIS; however, no information is available regarding the acute phase of NAIS.

The pivotal role of inflammatory pathways in ischemic brain injury is supported by recent years’ research data. T lymphocytes appear to play a central role in ischemic infarct development; they appear in the brain tissue within hours of the hypoxic insult and can be detected for up to a month after the injury. T cell deficiency has been shown to result in smaller infarct size and improved neurological outcome in murine models [33, 34]. Several cytokines have been connected to acute ischemic stroke in adults. Pro-inflammatory cytokines IL-1β, IL-8, MCP-1, TNF-α, and IFN-γ appear to exacerbate cerebral injury in adults, whereas anti-inflammatory cytokines such as TGF-β and IL-10 appear to be neuroprotective [35–37]. Although the immune system of neonates shows many differences compared to adults, the inflammatory network appears to play a similarly critical role in ischemic brain injury; therefore, it is reasonable to hypothesize that the same cytokines might also influence the course of neuroinflammation in the neonatal brain.

In this paper, we present four NAIS cases, in whom detailed analysis of intracellular and plasma cytokine levels are available from immediately after birth until the first month of age. To our knowledge, no such information is available in humans in NAIS; therefore, the data presented could serve as a basis for future, larger-scale case-control studies.

## Case presentation

### Patients and methods

All neonates were admitted to the regional cooling center with the initial diagnosis of HIE, requiring therapeutic hypothermia. Eligibility for cooling was assessed according to the TOBY criteria [38], and hypothermia was initiated between 1 and 5 h of life. Rectal temperature was maintained between 33 and 34 °C for the 72-h intervention period. Clinical or culture-proven sepsis was not detected in any of the infants. All infants received regular preventive intravenous antibiotics, i.e., ampicillin and gentamicin during the hypothermic treatment. MRI data were interpreted by radiologists who were blinded to the clinical status of the neonates, based on criteria defined by Rutherford et al. [39, 40].

A total of 33 term neonates were enrolled with written parental consent in a study focusing on inflammatory changes in HIE (approved by the Hungarian Medical Research Council (TUKEB 6578-0/2011-EKU)). Four infants were diagnosed with NAIS after their MRI scans were completed. One infant was excluded due to suspected metabolic disease. In the HIE group, data from 28 neonates were analyzed and divided into two groups based on the severity of the hypoxic-ischemic encephalopathy. The groups were determined based on the initial amplitude-integrated EEG (aEEG) recordings and the recovery time during monitoring [37] together with the MRI scans, which were performed within the first week of life. The severe group (*n* = 11) was formed of newborns with moderate-to-severe HIE signs on the MRI scan AND burst-suppression or continuous extremely low voltage or flat tracing background activity on aEEG OR normalization of aEEG after the 48th hour of life or never, OR early death (< 28 days). Neonates that met none of the above-listed criteria constituted the moderate group (*n* = 17) (mild HIE signs on MRI scans AND continuous or discontinuous normal voltage background activity on aEEG OR normalization of aEEG activity before the 48th hour of life) [41]. Pooled data from the cohort of four NAIS patients were compared to infants with HIE. In the severe HIE group, three infants deceased before 1 month of age due to the severity of the insult. Available data from these neonates were included at the relevant time points within the severe group. Therefore, 72-h, 1-week, and 1-month data were missing in the case of two infants and 1-month data were missing from one infant. Clinical characteristics of infants are summarized in Table 1.

**Table 1:** Clinical characteristics of neonates in the moderate and severe HIE and NAIS groups upon admission (within 12 h of age)

	Moderate HIE (*n* = 17)	Severe HIE (*n* = 11)	NAIS (*n* = 4)
Male gender (%)	10 (59%)	7 (64%)	2 (50%)
Birthweight (g)	3330 [2860–3605]	3000 [2490–3300]	3115 [2540–3885]
Gestational age (week)	39 [37–40]	38 [37–40]	39.5 [38.25–40]
No. of C-sections (%)	10 (59%)	8 (73%)	3 (75%)
Apgar at 1 min	3 [0.5–4.5]	1 [0–3]	4^b^ [3–5]
Apgar at 5 min	6 [5–7]	2^a, b^ [0–4]	5.5^b^ [5, 6]
Apgar at 10 min	7 [5–8]	4^a^ [1.75–5.25]	7^b^ [6, 7]
Worst pH	7.03 [6.87–7.12]	6.86 [6.62–7.06]	6.94 [6.88–7.00]
Worst BD (mmol/L)	18.05 [16.65–21.28]	20.4 [19.38–23.5]	14^b^ [14–17]
S100 (μg/L)	7.5 [2.33–28.85]	21.8 [3.8–30.0]	3.6^b^ [1.88–18.35]
LDH (U/L)	2072 [1371–5274]	3335 [1879–5792]	1874 [1676–3187]

Data are presented as median and interquartile range; a = moderate vs severe HIE, *p <* 0.05; b = severe HIE vs NAIS, *p <* 0.05

Two milliliters of venous blood was collected between 3 and 6 h of life (at admission), as well as at 24 h, 72 h, and 1 week of life, adjusted to blood sampling required by clinical care. A further venous blood sample was obtained at 1 month of age during a routine outpatient follow-up appointment.

### Flow cytometry

Plasma was separated from peripheral blood samples by centrifugation. Plasma samples were aliquoted and immediately frozen and stored at − 80 °C for later determination of cytokine concentrations.

Remaining cells were resuspended in RPMI (Roswell Park Memorial Institute)-1640 medium (Sigma-Aldrich, St. Louis, MO, USA). Cells were incubated with PMA (phorbol 12-myristate 13-acetate) (50 ng/ml), ionomycin (1 μg/ml), and BFA (Brefeldin A) (10 μg/ml) for 6 h at 37 °C to allow intracellular accumulation of cytokines. For surface marker staining, samples were then incubated with the following fluorochrome-conjugated anti-human monoclonal antibodies: CD4 PE-Cy7 (phycoerythrin-cyanine 7) and CD8 APC-Cy7 (allophycocyanin-cyanine 7) (panel 1) or CD4 APC-Cy7 and CD49d PerCP (peridinin-chlorophyll-protein) (panel 2), according to the manufacturers’ instructions (all from BioLegend, San Diego, CA, USA). Red blood cells were lysed and PBMCs were permeabilized using FACSLysing and FACSPermeabilizing solutions (BD Biosciences, San Jose, CA, USA). Cells were washed and resuspended in PBS (phosphate buffer saline) and divided into two equal aliquots and stained according to the manufacturers’ instructions for intracellular cytokines using the following conjugated anti-human monoclonal antibodies or the appropriate isotype controls: IL-6 PE (phycoerythrin), IL-17A PerCP, IL-10 APC (allophycocyanin), and IFN-γ FITC (fluorescein isothiocyanate) (for panel 1), or TNF-α PE-Cy7, FoxP3 PE, TGF-β APC, and IL-1β FITC (for panel 2), respectively (all from BioLegend). Following labeling, cells were washed and resuspended in PBS for flow cytometry analysis. Samples were analyzed immediately on a FACSAria flow cytometer (BD Biosciences) equipped with 488- and 633-nm excitation lasers. Data were processed using the FACSDiVa software (BD Biosciences). One hundred thousand cells were recorded. Evaluators of flow cytometry data were blinded to the clinical status of the neonates.

### Immunoassays

Plasma samples were stored at − 80 °C until analysis. The plasma levels of the following cytokines, chemokines, and growth factors were determined using Bio-Plex Pro Assays (Bio-Rad Laboratories, Hercules, CA, USA): IL-1β, IL-2, IL-4, IL-5, IL-6, IL-7, IL-8, IL-10, IL-12, IL-13, IL-17, IFN-γ, TNF-α, TGF-β, G-CSF, GM-CSF, MCP-1, MIP-1b, and VCAM. Bio-Plex Pro Assays are immunoassays formatted on magnetic beads that utilize principles similar to those of a sandwich ELISA. Capture antibodies against the biomarker of interest are covalently coupled to the beads. A biotinylated detection antibody creates the sandwich complex, and the final detection complex is formed by the addition of a streptavidin-phycoerythrin (SA-PE) conjugate, where PE serves as the fluorescent reporter. Reactions are read using a Luminex-based reader.

### Statistical analysis

Data are expressed as median and interquartile range. Comparisons between sample populations were performed with Mann-Whitney tests, as a test of normality (performed according to Kolmogorov-Smirnoff) indicated non-normal distribution of data. Comparisons between the paired values (samples collected at different time points) in the same population were made with Friedman tests. *p* values less than 0.05 were considered significant. Outliers were identified using Grubbs’ tests and were excluded from analyses. Statistics were calculated using the GraphPad Prism 5 software (La Jolla, CA, USA).

### NAIS case presentations

#### Case 1

The first patient was admitted following an emergency cesarean section performed due to oligohydramnios and fetal tachycardia on the 40th week of gestation. There was no maternal history of chorioamnionitis. However, the mother smoked during pregnancy. The amniotic fluid was stained with thick meconium. The neonate was born with an Apgar score of 5/6/7. She required intubation and ventilation at birth. Her first capillary blood gas showed lactic acidosis (pH 7.03, BE − 14 mmol/L, lactate 12.4 mmol/L), and she became irritable and had generalized increased muscle tone; thus, hypothermic treatment was initiated. On the second day of life, she presented with symptoms characteristic of pulmonary hypertension, which resolved after 1 day of NO inhalation and she was extubated on day 3 of life. Her early neurodevelopmental examination showed mild central hypotonia.

Cranial MRI with diffusion-weighted imaging was performed on day 4 of life, which showed a distinct, 5 × 7 mm area of ischemia with decreased diffusion in the left thalamus and no evidence of bleeding. MR spectroscopy showed a decrease in metabolites, correlating to minimal hypoxic-ischemic encephalopathy.

#### Case 2

The second neonate was admitted following an emergency cesarean section due to complete placental abruption on the 39th week of gestation. He was born with an Apgar score of 3/5/6/8. At the time of birth, he had a heartrate of 80/min, had no respiratory effort, and was hypotonic and pale. Following bag and mask ventilation, his heartrate normalized; however, only gasping could be observed, so he was intubated. He later became irritable. His initial blood gas showed severe metabolic acidosis (pH 6.99, BE − 14). A chest X-ray was performed, which showed a pneumothorax on the right side, which was drained and hypothermic treatment was initiated. Due to severe anemia and low blood pressure, he required blood transfusion on two occasions and inotropic support for 6 days, respectively*.* An early neurodevelopmental examination indicated grossly abnormal central and peripheral tone distribution*.*

Cranial MRI was performed on day 4 of life. Diffusion-weighted imaging showed a large area of ischemia with decreased diffusion in the area of the left middle cerebral artery, and several smaller lesions in the area of the right middle cerebral artery. The area of the left parietal cortex was blurred with hyperintensive signal subcortically*.* There were no signs of bleeding. TOF MRI angiography showed marked irregularity and significantly decreased blood flow in both, but particularly in the left MCA. No MRI signs of hypoxic-ischemic encephalopathy were present.

#### Case 3

The third neonate was born from an uncomplicated pregnancy on the 40th week of gestation, with an Apgar score of 5/5. The neonate was hypotonic and had no spontaneous breathing; therefore, she was intubated. The first gasping breaths were observed at 15 min after birth, but she remained generally hypotonic. The first blood gas showed severe lactic acidosis (pH 6.89, BE − 18 mmol/L, lactate 15 mmol/L). Therapeutic hypothermia was commenced. The initial aEEG recordings showed abnormal background activity; however, this normalized after a few hours. The early neurodevelopmental examination reported moderate generalized hypotonia.

Cranial MRI was performed on day 3 of life. Diffusion-weighted imaging showed a small (3 mm) ischemic lesion with decreased diffusion in the right thalamus. No other signs of ischemia and bleeding or signs of hypoxic ischemic encephalopathy could be observed.

#### Case 4

The fourth neonate was born from an uncomplicated pregnancy on the 38th week of gestation by emergency cesarean section due to imminent intrauterine asphyxia. He was born with an Apgar score of 3/6/7. He was flaccid with no signs of spontaneous breathing and a heart rate of 80/min. Following bag and mask ventilation, spontaneous breathing was noted after 5 min, but the neonate remained hypotensive and areflexive. He was intubated and hypothermic treatment was initiated. The aEEG recording showed no signs of seizures. The initial blood gas showed mixed acidosis (pH 6.875, BE − 14). The initial neurodevelopmental examination demonstrated abnormal central and peripheral tone distribution.

Cranial MRI was performed on day 1 of life. Diffusion-weighted imaging showed decreased diffusion indicating ischemia in the area of the right posterior cerebral artery. Smaller lesions were present in the right thalamus and the left parieto-occipital area. No signs of bleeding or hypoxic ischemic encephalopathy were present.

## Results

### Intracellular cytokine data

**Fig. 1:**
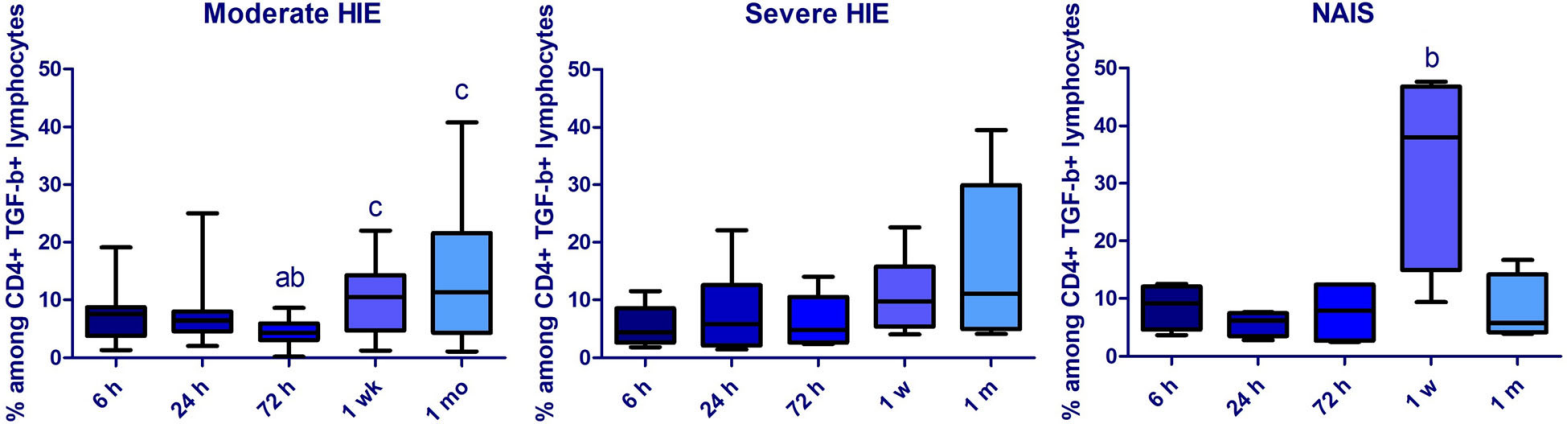
Alterations of the prevalence of CD4+ TGF-β + CD49d + cells within the CD4+ population in the first month of life in moderate (*n* = 17) and severe (*n* = 11) hypoxic-ischemic encephalopathy (HIE) and in NAIS (*n* = 4). Horizontal line: median, box: interquartile range, whisker: range. *p* < 0.05 a vs 6 h, b vs 24 h, c vs 72 h

At 6 and 72 h of age, the prevalence of CD8+ IL10+ lymphocytes remained lower in NAIS than in the severe HIE group. Although the prevalence of CD8+ IL10+ lymphocytes remained consistently lower in NAIS, the difference did not reach a significant level at the 24-h time point. On the contrary, the prevalence of CD4+ IL-10+ was higher at 24 h in NAIS compared to moderate HIE. The prevalence of IFN-γ + CD4+ cells showed an elevated tendency from 24 h in NAIS; however, this value did not reach significance, which could be due to the small population. At 1 week, the prevalence of TGF-β + lymphocytes prone to enter the CNS (CD4+ TGF-β + CD49d + lymphocytes) was elevated in NAIS compared to both HIE groups, and also compared to all other time points within the NAIS group (Fig. 1). On the other hand, at 1 month of age, the prevalence of CD4+ TGF-β + lymphocytes decreased in NAIS compared to HIE. Data are shown in Table 2.

**Table 2:** Intracellular cytokines—cell prevalence data

% of parent population		Moderate HIE (*n* = 17)	Severe HIE (*n* = 11)	NAIS (*n* = 4)
IL-10+ CD8+ / CD8+	6 h	2.75 (1.19–4.12)	4.19 (1.94–6.91)	1.10 (0.40–1.36) b, p = 0.01
IL-10+ CD4+ / CD4+	24 h	2.88 (1.56–7.03)	4.52 (3.02–6.59)	9.47 (5.10–20.68) a, p = 0.03
IL-10+ CD8+ / CD8+	72 h	3.51 (1.85–5.12)	5.30 (2.41–6.08)	1.39 (0.52–2.99) b, p = 0.03
TGF-β + CD49d + CD4+ / CD4+	1 week	10.54 (4.77–14.28)	10.0 (5.46–15.20)	38.00 (14.93–46.78) a, p = 0.03; b, p = 0.05
TGF-β + CD4+ / CD4+	1 month	3.51 (1.46–4.47)	3.54 (2.47–6.27)	1.30 (0.54–2.49) b, p = 0.05
IL-17+ CD8+ / CD8+	6 h	3.89 (5.26–14.40)	2.30 (1.73–4.49) c, p = 0.02	5.04 (4.62–7.68) b, p = 0.03
IFN-γ + CD4+ / CD4+	72 h	6.40 (3.70–11.80)	6.17 (4.75–13.01)	17.50 (2.74–30.20)
IL-6+ CD8+ /CD8+	72 h	4.37 (3.26–6.29)	5.63 (2.68–8.80)	8.87 (4.75–10.52)
IL-6+ CD8+ /CD8+	1 month	4.02 (2.40–10.74)	3.70 (2.31–6.16)	4.47 (2.88–8.33)

The prevalence of T cells expressing various cytokines are shown as the percentage of the parent population

At 6 h, the prevalence of CD8+ IL17+ lymphocytes was higher in NAIS than in severe HIE and CD8 cells expressed higher levels of IL-17 in NAIS than moderate HIE. The level of IL-1β in CD4+ cells was highest at 6 h and decreased significantly by 72 h within the NAIS group. The mean fluorescence intensity (MFI) of IFN-γ in CD4+ lymphocytes in NAIS was highest at 24 h and decreased significantly by 72 h, when it reached a lower level in NAIS than in either of the HIE groups. The MFI of IL-6 in CD8+ cells was higher at 72 h in severe HIE than in NAIS. Interestingly, by 1 month, the MFI of IL-6 in CD8+ decreased in both HIE groups and rose in NAIS. Data are shown in Table 3.

**Table 3:** Intracellular cytokines—mean fluorescence intensity (MFI) data

Arbitrary unit		Moderate HIE (*n* = 17)	Severe HIE (*n =* 11)	NAIS (*n =* 4)
MFI IL-17 / CD8	6 h	1427 (661.5–2402)	1591 (1157–7047)	4855 (2618–8706) a, p = 0.01
MFI IFN-γ / CD4	72 h	455 (149.5–770)	887 (495.5–1427)	42.25 (28.13–438.5) a, p = 0.04; b, p = 0.01
MFI IL-6 /CD8	72 h	701 (364.8–1441)	1073 (795–2017)	646.5 (188.7–736.3) b, p = 0.03
MFI IL-6 / CD8	1 month	429 (101.7–617.0)	347 (83–491)	918.5 (638.3–1347) a, p = 0.01; b, p = 0.02
MFI IL-10 / CD8	6 h	1883 (512.5–5817)	1874 (1157–3415)	705 (280–3313)
MFI IL-10 / CD4	24 h	674.5 (177.8–2608)	1084 (203–1892)	666 (242.5–1063)
MFI IL-10 / CD8	72 h	2167 (1462–5964)	1505 (1055–3819)	443.5 (371–5977)
MFI TGF-β / CD4	1 week	4620 (2654–6647)	3129 (1238–5621)	3260 (1452–3680)
MFI TGF-β / CD4	1 month	811.5 (253–1740)	634 (259–1789)	330.5 (123–2283)

The intracellular level of certain cytokines in T cells is shown by the MFI of each cytokine. Data are expressed as median and interquartile range; a = moderate HIE vs NAIS, *p <* 0.05; b = severe HIE vs NAIS, *p <* 0.05

We found no differences between NAIS and HIE in the intracellular production of IL-1β and TNF-α in CD4 or CD8 cells; however, these cytokines appear to be important in differentiating between the mild and severe form of HIE [41].

### Plasma cytokine data

**Fig. 2:**
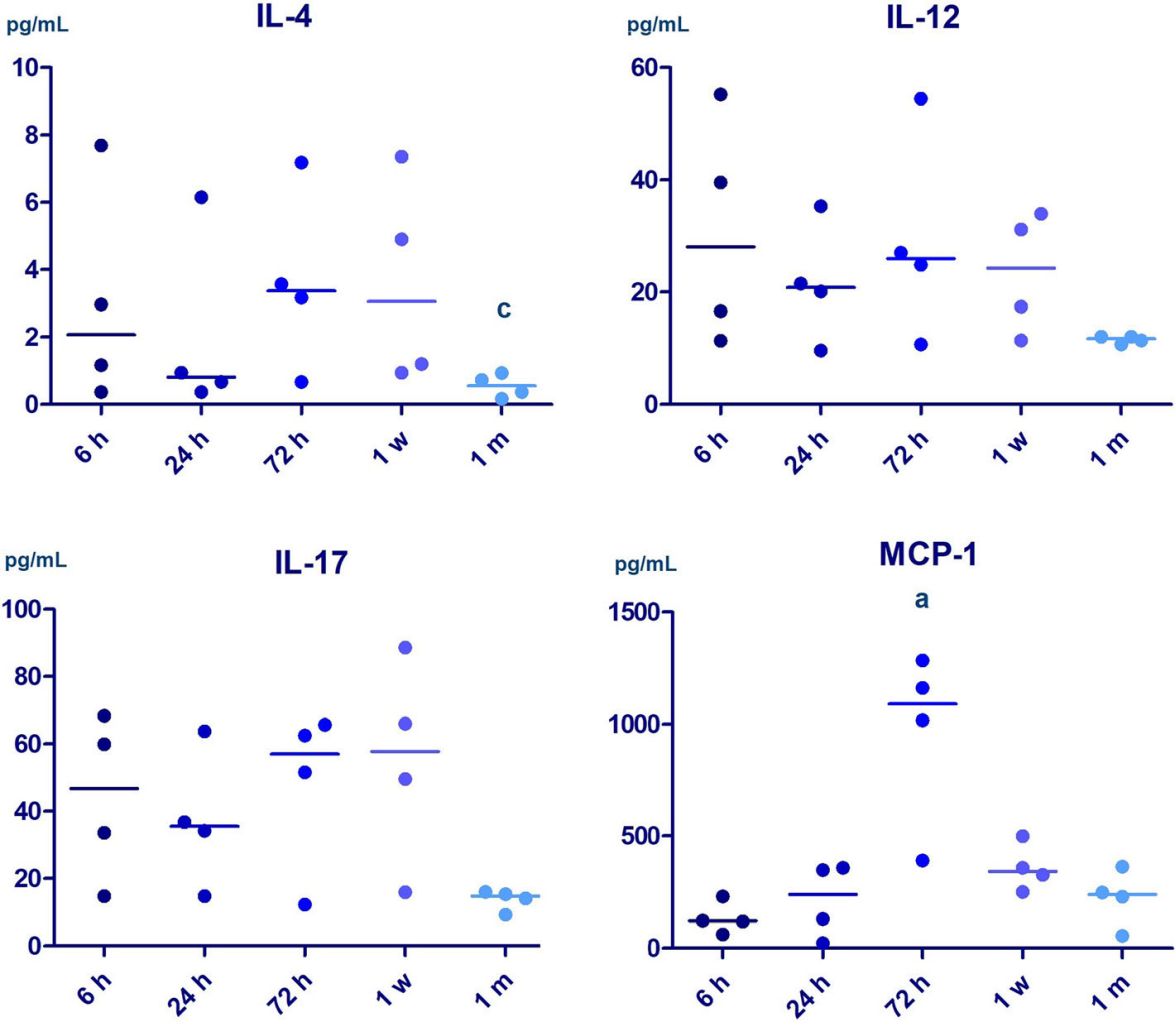
Alterations of plasma cytokine levels in the first month of life in neonatal arterial ischemic stroke (*n* = 4). Horizontal line: median. *p* < 0.05 a vs 6 h, b vs 24 h, c vs 72 h

At 72 h, we found a marked inflammatory response in NAIS, characterized by elevated plasma levels of IL-5, IL-17, and MCP-1 compared to HIE. Plasma MCP-1 level was the highest at 72 h in NAIS. By 1 month, however, inflammatory response appears to be decreased in NAIS compared with HIE, indicated by decreased plasma levels of IL-4, IL-12, and IL-17 (Fig. 2). The level of IL-4 was lower at 1 month than 72 h in NAIS.

We found no alterations in NAIS in the plasma level of IL-1β, IL-2, IL-6, IL-7, IL8, IL-10, IL-13, IFN-γ, TNF-α, TGF-β, G-CSF, GM-CSF, MIP-1b, and VCAM when compared with HIE. Data are shown in Table 4.

**Table 4:** Plasma cytokines

pg/mL		Moderate HIE (*n =* 17)	Severe HIE (*n =* 11)	NAIS (*n =* 4)
IL-5	72 h	1.37 (0.00–4.69)	0.20 (0.00–0.46)	3.42 (1.42–6.35) b, p = 0.01
IL-17	72 h	30.44 (26.65–51.62)	33.91 (29.16–48.02)	62.52 (51.62–645.7) a, p = 0.04, b, p = 0.05
MCP-1	72 h	251.9 (87.12–595.6)	678 (214–2311)	1090 (547.2–1254) a, p = 0.03
IL-4	1 month	1.68 (0.84–2.54)	1.52 (0.84–2.44)	0.55 (0.22–0.89) a, p = 0.04 b, p = 0.05
IL-12	1 month	18.11 (13.34–39.53)	21.52 (15.96–36.46)	11.74 (10.90–12.06) a, p = 0.05 b, p = 0.05
IL-17	1 month	32.58 (22.02–51.92)	31.11 (19.50–74.08)	14.75 (10.49–15.85) a, p = 0.02 b, p = 0.004

The plasma levels of cytokines are shown at different time points. Data are expressed as median and interquartile range; a = moderate HIE vs NAIS, *p <* 0.05; b = severe HIE vs NAIS, *p <* 0.05

## Discussion

The contribution of T lymphocytes to ischemic brain injury now appears to be clear, with CD8+ and CD4+ T cells appearing in the CNS already a few hours after an ischemic insult. The accumulation of T cells peaks around 3–4 days following injury, and the inhibition of T cell trafficking to the CNS decreases the deleterious consequences of neuroinflammation [42, 43]. Data from murine stroke models showed persisting presence of T cells up to 7 weeks after the ischemic attack [44]. In our NAIS patients, CD8 lymphocytes appear to show a different cytokine production pattern than HIE with a more prominent pro-inflammatory response in NAIS. At 6 h, CD8 cells from NAIS patients produce higher levels of pro-inflammatory IL-17 than those from neonates with moderate HIE, and CD8+ IL17+ cells are more prevalent in NAIS than in severe HIE. At 72 h, CD8 cells of neonates with severe HIE produce higher levels of pro-inflammatory IL-6 than CD8 cells of NAIS patients. IL-6 is known to be an important factor in the early phase of HIE, and this difference could reflect an aggravated neuroinflammation in severe HIE. On the other hand, IL-6 has also been described to have neurotrophic, neuroregenerative, and anti-inflammatory properties within the brain [45–48]. The alteration in intracellular levels of IL-6 in CD8 cells becomes reversed by 1 month, when it remains elevated in NAIS, whereas it decreases in both HIE groups, indicating that a sustained level of IL-6 might be present for a longer time in NAIS, which is in line with literary data [32]. Therefore, IL-6 may exert its neuroprotective effects over a longer period of time in NAIS.

Plasma cytokine levels also indicate a difference between NAIS and HIE, with a higher level of pro-inflammatory response at 72 h in NAIS than in HIE. We found elevated IL-5, IL-17, and MCP-1 levels at 72 in NAIS (see Table 3 for details.) MCP-1 was found to be elevated following ischemic insult after in utero LPS exposure in a chorioamnionitis associated perinatal stroke murine model [5]. We found an elevation in the level of MCP-1 at 72 h compared to 6 h value in NAIS and to moderate HIE, which supports previous data emphasizing the role of in utero inflammation. Both the plasma levels of IL-17 and the prevalence of pro-inflammatory Th17 and γδ T cells are known to increase 1 week after adult acute ischemic stroke [49] and decrease by 1 month. In line with these findings, we found a higher level of IL-17 in the second phase (at 72 h) of neuroinflammation in NAIS compared with HIE. By 1 month, however, the level of IL-17 decreased in NAIS, while it remained elevated in HIE. We observed the same phenomenon with other cytokines such as IL-4 and IL-12. Overall, the plasma levels of several cytokines appear to decrease by 1 month of age in NAIS and appears to remain at a higher level in HIE.

We also found alterations in the anti-inflammatory pathways. IL-10 was thought to be primarily produced by CD4+ lymphocytes; however, Trandem et al. demonstrated in a murine model of virus encephalitis that the most highly activated and cytotoxic CD8+ T cells are also an important source of IL-10 at the peak of the inflammatory response. Their data suggests that CD8+ cells autoregulate their activation by IL-10 production when the inflammatory response reaches a certain level and that CD8-derived IL-10 has a primarily local effect to prevent tissue damage. IL-10 expression by CD8 cells rapidly decreased after 7 days, while CD4+ cells continued to express IL-10 for at least 42 days [50]. Our data showed a significantly higher prevalence of IL-10+ CD8+ lymphocytes in severe HIE than NAIS in the first 72 h of life. IL10-producing CD8 cells were also more prevalent in mild HIE than in NAIS; however, the difference was not significant. A lower prevalence of IL-10-producing CD8 cells, which at the same time produce more of pro-inflammatory cytokines such as IL-17, could lead to a more prominent pro-inflammatory and cytolytic response with decreased auto-regulatory IL-10 production in CD8 cells in NAIS in the first 72 h. Another interpretation could be that the decreased level of IL-10 production by CD8+ cells reflects an in utero inflammatory response that decreases by the time of birth. Supporting this hypothesis, in NAIS, we found elevated prevalence of IL10+ CD4+ lymphocytes, which are known to produce IL-10 for over a month following the insult. Altogether, it appears that IL-10 production is different in T cell subpopulations in NAIS and HIE and the manipulation of the IL-10 pathway could be interesting to explore from a neuroprotective point of view.

The prevalence of TGF-β-producing CD4+ cells, the majority of which are regulatory T (Treg) cells, was also different in NAIS and HIE. TGF-β is one of the most important cytokines regulating CNS repair. We found increased CD49d expression in this population in NAIS at 1 week compared to 24 h and both HIE groups, reflecting a higher capacity of these cells to enter the CNS. Meanwhile, the prevalence of the entire population decreased by 1 month in NAIS compared to HIE. This appears to be in line with plasma cytokine data, which showed an increased inflammatory response in NAIS in the second phase of neuroinflammation which could be followed by an earlier reparative phase and a decreasing inflammatory response by 1 month of age

Therapeutic hypothermia is currently the standard of care for term neonates with HIE. There is substantial evidence supporting the role of hypothermia in reducing mortality and improving neurological outcome [51] in infants with hypoxic ischemic encephalopathy. There is notable evidence in animal focal cerebral ischemia models supporting the role of hypothermia in the reduction of infarct size [52] and data suggesting that hypothermia might reduce the risk of seizures after perinatal stroke and thus improve outcome [53]. Therefore, there is intense discussion in the scientific community about extending guidelines to include new categories for therapeutic hypothermia such as preterm HIE and perinatal stroke; however, many issues remain to be addressed. Based on results from our previous study, one of the mechanisms behind the neuroprotective effect of hypothermia may be the reduction of systemic inflammatory response, seen in the reduced level of cytokines such as IL-6 and IL-4 in HIE patients treated with hypothermia compared to the normothermia group [54]. Therefore, it is likely that hypothermia may similarly reduce cytokine levels following NAIS.

The main limitation of these data is the small number of cases; however, they could provide a hypothesis for future, larger case-control studies. Another limitation is the lack of healthy control population; however, due to ethical considerations and the large number of blood sampling, enrolling healthy neonates was not possible. We also saw a notable variation in certain plasma cytokine levels, which is likely a result of the relative instability of plasma cytokine levels, which was the basis of primarily focusing on intracellular cytokine production in the current study. The value of the data from this study could lie in providing incentive for further clinical research on factors enabling differentiation between the two disorders which often present with similar symptoms at birth. Gaining a better understanding of the underlying pathophysiological factors is a main aim at the current stage of research in NAIS.

## Conclusions

CD8 lymphocytes appear to show a shift in the pro-inflammatory direction in NAIS compared with HIE, with elevated production of pro-inflammatory IL-17 at 6 h, decreased auto-regulatory IL-10 production in the first 3 days, and elevated production of IL-6 at 1 month. Altogether, the inflammatory response appears to be higher at 72 h in NAIS than in HIE, but appears to decrease faster, indicated by lower levels of inflammatory markers at 1 month in NAIS than in HIE. Our data indicate similarities to observations in adult AIS and also support the hypothesis of an ongoing in utero inflammation prior to NAIS. Further clinical research in this area is pivotal, and a better understanding of the pathophysiology could lead to identifying specific factors to distinguish HIE from NAIS early and could open up new pathways for prevention and individualized care.

## Abbreviations

ACA: Anterior cerebral artery
aEEG: Amplitude-integrated electroencephalography
AIS: Acute ischemic stroke
BFA: Brefeldin A
HIE: Hypoxic ischemic encephalopathy
MCA: Middle cerebral artery
MFI: Mean fluorescence intensity
NAIS: Neonatal arterial ischemic stroke
PCA: Posterior cerebral artery
PMA: Phorbol 12-myristate 13-acetate
Treg: Regulatory T cell
